# The burden of rheumatic heart disease among children in Lagos: how are we fairing?

**DOI:** 10.11604/pamj.2018.29.150.12603

**Published:** 2018-03-14

**Authors:** Barakat Adeola Animasahun, Akpoembele Deborah Madise Wobo, Adejumoke Yemisi Itiola, Motunrayo Oluwabukola Adekunle, Olusola Yejide Kusimo, Fidelia Bode Thomas

**Affiliations:** 1 Department of Paediatrics and Child Health, Lagos State University College of Medicine, Ikeja, Lagos, Nigeria; 2 Department of Paediatrics, Jos University Teaching Hospital, Lagos, Nigeria

**Keywords:** Rheumaric, heart, disease, children, Nigeria

## Abstract

**Introduction:**

Rheumatic heart disease still remains a cause of morbidity and mortality in low and middle income countries, despite its eradication in developed societies. The study aimed to document the features of children with rheumatic heart disease using clinical evaluation and echocardiography and compare it with reports from other part of the country.

**Methods:**

A review of a prospectively collected data of patients with rheumatic heart disease who had echocardiography done from April 2007-Dec 2016. Information obtained from patients include age, sex, clinical indication for echocardiography, echocardiographic characterization of the valvular lesions and associated complications.

**Results:**

A total of 324,676 patients were seen at the Paediatric unit of LASUTH from 2007 to 2016, out of which 36 had Rheumatic heart disease. This translates to a prevalence of 1.1 per 10,000 patients who presented at the study site during the study period. The prevalence of RHD amongst all the patients with structural heart disease was 2.6%. The mean age of patients was 9.12 ± 2.75 years with a male to female ratio of 1.6: 1. The most common valve affected was mitral valve. Heart failure was the most common mode of presentation found in 91.6%. Other complications were pulmonary hypertension and pericardial effusion.

**Conclusion:**

Rheumatic heart disease is still prevalent among children in Lagos although the prevalence is reducing. Heartfailure is the commonest mode of presentation and complication in them.

## Introduction

Rheumatic heart disease (RHD) is a non-suppurative complication of group A Beta hemolytic streptococcal throat infection. It affects children and young adults in developing countries and most of these patients presents in heart failure and require surgical intervention [1]. It is estimated that 12 million people worldwide are affected by rheumatic fever and rheumatic heart disease and two-thirds of these are children between ages of five and fifteen, with 79% of cases from developing countries particularly those in the African continent [2]. Rheumatic heart disease is one of the most common form of acquired cardiovascular disease, in Sub Saharan Africa. According to a World Health Organization (WHO) estimate over a decade ago, RHD affects children of school going age with a prevalence of 5.7 cases per 1000 school children.1 The prevalence varies from one region to another but it is known that the rates are still high in sub-Saharan Africa compared with the Western countries [3]. A systematic review conducted in South Africa revealed a high prevalence of RHD, with up to 20.2 per 1,000 children with asymptomatic RHD in some regions in that country [4]. In Nigeria, the prevalence rates varies from region to region with some centres reporting rates of 12.4 per 1,000 children seen in the hospital [5]. The new clinical criteria of 2012 for the diagnosis of ARF/RHD classified ARF into definite initial episode of ARF, definite recurrent episode of ARF in a patient with known past ARF or RHD and probable ARF (first episode or recurrence) [6]. Proven preventive strategies including the use of prophylaxis for rheumatic fever and socio-economic improvement was recognized over thirty years ago [7]. However, in developing countries, social determinants of the disease such as adequate housing, access to primary health care, education and availability of cardiologic diagnostic tools and cardiac surgery are still a major challenge [8]. The decline in the prevalence of rheumatic heart disease in developed countries has been attributed to high standard of living and access to medical care [8]. There is no doubt that rheumatic heart disease still remains a cause of morbidity and mortality in low and middle income countries, despite its eradication in developed societies. This study presents the distribution or rheumatic valvular lesions as seen at echocardiography in the Paediatric cardiology unit of the Lagos State University Teaching Hospital (LASUTH) over a ten period (from January 2007 to December 2016).

## Methods

**Study setting**: The study was conducted at the Lagos State University Teaching Hospital, (LASUTH) Ikeja. A 600 bedded Urban Tertaiary Centre in Lagos State, Westen Nigeria which serves as a refering centre for not only more than twenty general hospitals in Lagos, but also private hospitals and federal medical centre in Lagos. It receives patient from South Western Nigeria and from all over the country especially Paediatric patients due to the free health policy for the under twelve years of age.

**Subject recruitment and data collection**: The present study is a review of a prospectively collected data of patients diagnosed with Rheumatic Heart disease using clinical evaluation and echocardiography at the Paediatric Cardiology unit between January 2007 and December 2016. All patients had chest radiograph, electrocardiography and echocardiographic evaluation. Anti-streptolysin O antibody was assayed on all patients with a strong suspicion of acute rheumatic fever. The age, sex, clinical indications and echocardiographic characterization of valvular lesions and associated complications of the patients were documented.

**Case definition of RHD and pulmonary artery hypertension**: Rheumatic heart disease was defined by the presence of any definite evidence of valve regurgitation or stenosis seen in two planes on Doppler examination and at least two morphologic abnormalities such as restricted leaflet mobility, focal or generalized valvular thickening and abnormal sub-valvular thickening of the affected valves [9]. Pulmonary artery hypertension (PAH) was identified using a combination of ECG and transthoracic echocardiography (Two-dimensional and Doppler) [10]. ECG findings include evidence of right ventricular dilatation and hypertrophy. Two-dimensional features include; increased thickness of the right ventricle, paradoxical bulging of the septum into the left ventricle during systole, right ventricular dilation, right atrial dilatation and or tricuspid regurgitation. Doppler echocardiography was used to measure the pulmonary artery pressure by means of tricuspid regurgitation velocity measurements. Pulmonary pressure greater than 25mmHg at rest is diagnostic of PAH [11]. The severity of PAH is classified as mild (PAH from 25-40mmHg), moderate (PAH from 41 to 55mmHg) and severe (PAH > 55mmHg) [12]. Cardiac catheterization is not routinely done on all patients and thus diagnosis of PAH was not made with cardiac catheterization.

**Data analysis**: Data were analyzed using Statistical Package for Social Sciences (SPSS) version 20.0. The frequencies of each valvular lesions, clinical indications for echocardiography, male to female ratio and mean age at diagnosis of the patient were documented. Mean, standard deviation and other parameters were generated as necessary for continuous data. Means of continuous variables were compared using the Student t test and proportions using Chi-square test. Level of significance set at p < 0.05

**Ethical consideration**: Consent was obtained for Echocardiography from the parents of the children. In the data storage and presentation, no personal information was used unlawfully. The details of each subject was made confidential.

## Results

**Prevalence of RHD**: A total of 1,846 echocardiographs were performed in the study period, out of which 1364 had structural heart diseases (congenital and acquired). Rheumatic valvular disease was documented in 36 of those patients. The prevalence of RHD amongst all the patients with structural heart disease was 2.6%. There were 136 cases of acquired heart diseases in the period under review, the prevalence of RHD amongst those with acquired heart disease (AHD) was 26.5%. Within the study period, a total of 324,676 children were seen in the Department of Paediatrics. They comprised of 173,695 males and 150,981 females. The prevalence of RHD amongst the children who presented to the study center during the period of study was 1.1 per 10, 000 children. Table 1 shows the prevalence of RHD in Nigeria in the last two decades.

**Table 1:** Literature extraction of Nigeria prevalence of RHD in the last two decade

	Author/Year of publication	StudyPeriodInMonths	Total nowith RHD	NowithAHD	NOwithSHD	Total adm	M:F	Age rangein yrs	Prevalence in AHD	Prevalence in SHD
Lagos(South-West)	Present study	120	36	136	1364	324,676	1.7:1	4-13	26.5	2.6
Lagos/Benin/Abuja(multi-location)	Sadoh et al /2014	42	23	132	NA	NA	1:1.5	5-17	17.4	NA
Lagos(South-West)	Okoromah et al /2008	48	12	42	270	26,568	?	?	28.6	4.4
Ibadan (South-West)	Adebayo et al /2016	12	6	18	210	NA	NA	5-14	33.3	2.9
Abeokuta(South-West)	Ogah et al /2014	60	107	NA	NA	NA	1:1.6	3-92	NA	N/A
Port-Harcourt(South-South)	Akpa et al /2012	12	32	NA	NA	NA	1.4:1	17-65	N/A	N/A
Jos(North-Central)	Bode-Thomas et al /2013	120	101	175	564	NA	1:1.4	1-18	57.7	17.9
Zaria(North-Central)	Danbauchi et al /2004	36	47	NA	7600	NA	1.4:1	5-52	NA	7.8
Kano(North-West)	Sani et al /2007	4	129	NA	1312	NA	1>1.7	5-60	NA	9.8
Sokoto(North-West)	Sani et al /2015	60	47	110	NA	3810	1:1.5	4-15	42.7	NA

No-Number NA-Data not available RHD-Rheumatic heart disease

AHD-Acquired heart disease

SHD-Structural heart disease

Adm-Admission

**Demographic characteristics of the study subjects**: Of the 36 patients with RHD, there were 22 males and 14 females with a male to female ratio of 1.6:1. The children were aged 4 to 13years. Most of the patients, (50%) were between five and 10 years of age at diagnosis with a mean age of 9.12 ± 2.75 years. The mean age of the males and females were 9.24 ±2.14 and 8.96 ± 3.52. There was no significant difference in the both the mean age and the age distribution of both gender; p = 0.619 and 0.591 respectively. The Table 2 depicts the gender and age distribution of the patients.

**Table 2:** Gender and age distribution of the patients

Age group (years)	Male	Female	Total (%)	P value
1-4.9	1	2	3 (6.5)	0.840
5-10	12	6	18 (51.6)	
>10	7	6	13 (41.9)	
Median	9.1	10	9.12	

The ages of 2 children

**Valvular lesions**: The most common valve affected in the patients was the mitral valve with mitral regurgitation, which occurred alone or combination with mitral valve prolapse. Of the 36 patients with RHD, 20 (62.5%) had tricuspid regurgitation from pulmonary artery hypertension. The valvular lesions are depicted in Table III. The most common valve affected was mitral (95%), while the least affected was Aortic (7.5%). Half of the patient (50%) had mitral valve affectation with tricuspid regurgitation from pulmonary artery hypertension as depicted in Table 3.

**Table 3:** Pattern of valvular lesions in RHD

Pattern of valvular lesions	Frequency	%
Isolated MVP/MVR	17	42.5
Isolated AR	1	2.5
Isolated MS	1	2.5
MVP with AR	1	2.5
MVR with TR	19	47.5
AS, MVP, TR	1	2.5

**Figure 1:**
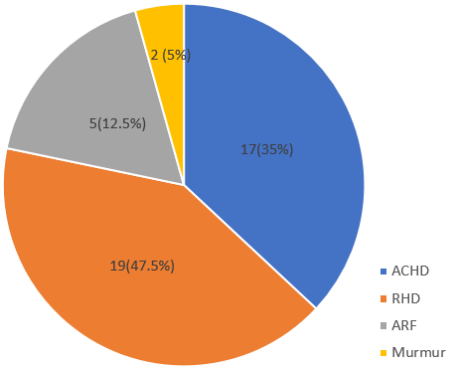
Indication for electrocardiography

**Pre-echocardiographic diagnosis and complications**: All the patients were referred from both within and outside the hospital. The reason for referral and cardiac evaluation are depicted in Figure 1. The most common was a clinical diagnosis of RHD followed by a suspicion of an Acyanotic Congenital Heart Disease (ACHD). Other indications include, acute rheumatic fever and a murmur. The complications encountered were heart failure, mild, moderate and severe pulmonary hypertension and cardiomegaly. The most common of the complications was congestive cardiac failure which occurred in half of the patients. This was closely followed by pulmonary artery hypertension. Table 4 depicts the complications in the study subjects. Table 5 shows the echocardiographic parameters of the subjects. There was reduced mean left ventricular ejection fraction (53.6), increased mean left ventricular mass index (27.1g), and increased left atrial diameter (39.2mm) among the subjects.

**Table 4:** Complications in the study subjects

Complication	Number of patients	%
CCF	27	51.0
PAH		41.5
Mild	15	
Moderate	4	
Severe	3	
Cardiomegaly	4	7.5

CCF- Congestive cardiac failure; PAH- Pulmonary artery hypertension

**Table 5:** Shows echocardiographic features of the study subjects

Features	Range	Mean±SD
LVDd	40-64	50.5±8.5
LVDs	6-40	27.1±14.6
IVSd	6-9	7.3±
PWd	4-11	8.5±3.8
Ejection fraction	20-68	53.6±19.2
Fractional shortening	9-37	28±11.0
Aortic root diameter	18-30	22±8.5
Left Atrial diameter	26-53	39.2±10.8

## Discussion

Rheumatic heart disease is one of the most common and preventable acquired heart disease with valvular heart damage as the hallmark [5]. While the number of cases in developed countries have reduced drastically, the same cannot be said of developing countries like those in sub-Saharan Africa. The prevalence of RHD among children in the present study was 2.6 and 26.5% in structural and acquired heart diseases respectively. The general trend noted in Nigeria from the literature is 2.9 to 9.8% and 17.4 to 57.7% for the prevalence amongst the structural and acquired heart diseases respectively. The values documented in the present study is thus within the reported range in Nigeria [5, 13-16]. In the present study, the hospital based population prevalence of RHD was 1.1 per 10,000 children. This was slightly lower than an earlier report by Okoromah et al, [14] within the same location almost a decade ago and significantly lower than that by Sani and Colleagues [5] from the North Western region of Nigeria. The range of values in Nigeria is wide and possible explanation lies in the different geographic locations where the research was carried out and heterogeneity of the sample methods in the various studies as depicted in Table 1. It was observed that the prevalence appears to be lower in South-Western Nigeria compared to the Northern parts of the countries. The reason is not presently clear but possible explanation may be adduced from a more improved living conditions, increased access to antibiotics for throat infections and improved access to health care in the southern parts of the country. The health indices in the country is also skewed with much better indices in the south compared to the north [17]. All of these may be responsible for the lower prevalence of RHD in the South. The heterogeneous nature of the method among the studies is also responsible for the wide range of prevalence values. For example, Sani et al16 and Danbauchi et al [18] both included adult population in their study, this may have been responsible for a higher prevalence of RHD in those studies. Furthermore, the duration of the studies varies from one study to another, while some studies were over a 12-month period, others were 5-10years. This may have also contributed to the varying prevalence in the country. The prevalence of RHD in the present study is lower than the documented prevalence in other countries in Africa. The prevalence rate in a recent study in Kenya was 22.1% and 50.4% for structural and acquired heart diseases respectively [19]. Similarly, a study in Cameroon documented a prevalence of 41.1% among the acquired heart diseases [20]. The values are lower in the present study compared to those reported in other African studies because of the regional differences in the pattern of RHD in countries in Africa. Despite the relatively lower values documented in the present study, rates of RHD are known to be high in Africa.

According to a WHO report, Africa is one of the regions with the highest prevalence of RHD and the rate doesn't appear to be decreasing compared to regions like the European countries where it is almost extinct [3]. The reasons adduced for the higher rates includes the availability of major advances in medical and surgical treatment with improved survival and that RHD is more rigorously sort out by echocardiography. Given the lower prevalence of RHD in Southern Nigeria compared to the north, it is imperative that health policies and practices be scaled up in the northern part of the country to reduce the burden therein. The mean age at diagnosis in the present study, is consistent with reports from other studies [5, 13,16,20, 21]. Most of the children with RHD were over 5 years of age and majority were between 5-10years of age. It has been shown that RHD is more common in children between 5-15years. In the present study, there were more males than the females with RHD. The finding in this regard is in contrast with most reports where there is a female predominance in both adult and children [5,13,16,22,23]. However, few studies have reported a male predominance [16, 24,25]. The reason for the female predilection in most of the studies is not known. Given that most of the studies are hospital based, not all the patients with the disease are captured. A community based study will present data that will be more representative of the demographics of RHD. In the present study, Mitral regurgitation was the most common valvular lesion occurring either alone or in combination with other valvular lesions. This finding mirrors reports from previous studies [5,13,19]. It has been shown from previous reports that mitral valvular disorders are more common compared to the other valvular lesions. However, the reason for the overwhelming predisposition of mitral valve is yet to be unraveled [26]. Tricuspid regurgitation was second most common valvular lesion seen. It was documented in half of the patients. This finding is not out of place because pulmonary artery hypertension (PAH) is a common complication of RHD and severe forms of PAH will result in tricuspid regurgitation [19,27]. In contracts to the regurgitant lesions, stenotic valvular lesions were only seen in two patients. One patient had a mitral stenosis and another with aortic stenosis. The finding in this regard is not out of place given that stenotic valvular lesions depicts advanced or chronic RHD which is not seen until late adolescents and adulthood. Rheumatic heart disease is a major cause of morbidity and mortality in affected children. Heart failure and pulmonary artery hypertension were the most common complications observed in the patients. This finding is in keeping with reports from a previous studies [19,22,27].

Heart failure results from valvular insufficiency or stenosis. Given that majority of the patients had mitral insufficiency, it was not surprising that heart failure was a common morbidity amongst them. Similarly, PAH results from severe mitral valvular lesions [19,28]. The finding of pulmonary hypertension in half of the patients is thus in keeping with the prevalent valvular lesions amongst them. The implication of this is that common co-morbidities such as heart failure and PAH should be anticipated and managed promptly in all patients with RHD. In a review of predictors of mortality in chronic rheumatic heart disease, Talwar and Gupta [29] noted that, the severity of valvular damage and it's haemodynamic consequences to which PAH and heart failure belongs, is a major factor attributable to mortality in RHD. Other morbidities such as arrhythmia and infective endocarditis are consequence of severe valvular disease that have been associated in the mortality of patients with RHD, but those were not documented in our subjects. Rheumatic heart disease is a major public health problem in Nigeria and most developing countries. This is because these countries are still burdened with poverty, low level of awareness, poor health seeking behaviour and inadequate treatment. These factors result in persistence of the disease and a poorer outcome [25]. The huge strides achieved in the developed regions of the world in diagnostic options, surgical and interventional management of heart disease has not been replicated in Africa [30]. The diagnostic challenge has reduced in a few centers due to the availability of echocardiography imaging facilities, but this has not been matched with the availability of cardiac surgeries. It has been recommended that primary and secondary prevention of rheumatic fever should be strengthened at all levels of care [31, 32]. This includes prompt use of penicillin prophylaxis in the vulnerable age group, as well as early treatment of streptococcal sore throat which precludes the development of Rheumatic fever and Rheumatic Heart Disease. In addition, long term treatment of rheumatic fever can halt the disease progression [25]. Furthermore, emphasis should be placed on programs such as early case finding and prompt diagnosis of children with rheumatic heart disease, provision of adequate medical services of high quality in hospitals and a good follow up care [8].

## Conclusion

In conclusion, there has been a decline in the prevalence of Rheumatic heart disease among children in Lagos in the last decade. Males were more commonly affected than females. Heart failure and pulmonary hypertension were the commonest complications and Mitral valves are the most commonly affected valve.

### What is known about this topic

- Rheumatic heart disease is an important cause of morbidity and mortality among children in the African Sub region;
- It is almost non-existent in the developed countries since the advent of penicillin;
- Study done in a populous city is likely to be more representative of the pattern in the developing world.

### What this study adds

- Rheumatic heart disease is still prevalent among children in Lagos;
- The prevalence of rheumatic heart disease among children in the southern part of Nigeria is lower than those from the northern part of Nigeria;
- The prevalence of rheumatic heart disease among children in the southern part of Nigeria is reducing.

## References

[1] WHO Rheumatic fever and rheumatic heart disease. Report of a WHO Expert Consultation.

[2] Mackay J, Mensah G, Prevention WHO and C for DC and P (2004). Rheumatic Fever and Rheumatic Heart Disease. The Atlas of Heart Disease and Stroke.

[3] Press D (2011). The worldwide epidemiology of acute rheumatic fever and rheumatic heart disease.

[4] Zuhlke L, Engel M, Watkins D, Mayosi B (2015). Incidence, prevalence and outcome of rheumatic heart disease in South Africa: a systematic review of contemporary studies. Int J Cardiol.

[5] Sani UM, Ahmed H, Jiya NM (2015). Pattern of acquired heart diseases among children seen in Sokoto, NorthWestern Nigeria. Niger J Clin Pract.

[6] RHD Australia (2012). ARF RHD Guideline. The Australian guideline for the prevention, diagnosis and management of acute rheumatic fever and rheumatic heart disease.

[7] Jaiyesimi F (1982). Chronic rheumatic heart disease in childhood: Its cost and economic implications. Trop Cardiol.

[8] Wallace H (1955). Changing status of rheumatic fever and rheumatic heart disease in children and youth. Am J Dis Child.

[9] Snider R, Snider R, Serwer G (1997). The normal echocardiograph examination. Echocardiography in Paediatric haert disease.

[10] Jone PN, Ivy D (2014). Echocardiography in pediatric pulmonary hypertension. Front Pediatr.

[11] Rosenzweig EB, Widlitz AC, Barst RJ (2004). Pulmonary arterial hypertension in children. Pediatr Pulmonol.

[12] Khan MG, Khan MG, Lynch JP III (1997). Pulmonary hypertension and co-pulmonale. Pulmonary disease diagnosis and therapy: a practical approach.

[13] Sadoh E, Uzodimma C, Daniels Q (2014). Childhood acquired heart disease in Nigeria: an echocardiographic study from three centres. Afr Health Sci.

[14] Okoromah CAN, Ekure EN, Ojo OO, Animasahun BA, Bastos MI (2008). Structural heart disease in children in Lagos: profile, problems and prospects. Niger Postgrad Med J.

[15] Adebayo BE, Ogunkunle OO, Omokhodion SI, Luke RD (2016). O riginal Article The spectrum of structural heart defects seen in children at the University College Hospital, Ibadan. Nig J Cardiol.

[16] Bode-Thomas F, Ige O, Yilwan C (2013). Childhood acquired heart disease in Jos, North central Nigeria. Nig Med J.

[17] Wollum A, Burstein R, Fullman N, Dwyer-lindgren L, Gakidou E (2015). Benchmarking health system performance across states in Nigeria: a systematic analysis of levels and trends in key maternal and child health interventions and outcomes 2000-2013. BMC Med.

[18] Danbauchi SS, Alhassan MA, David SO, Wammanda R, Oyati IA (2004). Spectrum of rheumatic heart disease in Zaria. Ann Afr Med.

[19] Kironget A, Kiptoon P, Koech M (2014). Echocardiographic characteristics of children with rheumatic heart disease at MOI Teaching And Referral Hospital (MTRH), Eldoret, Kenya. Eur J Biol Med Sci Res.

[20] Nkoke C, Menanga A, Boombhi J, Chelo D, Kingue S (2015). A new look at acquired heart diseases in a contemporary sub-Saharan African pediatric population: the case of Yaoundé, Cameroon. Cardiovasc Diagn Ther.

[21] Asani MO, Sani M, Karaye K, Adeleke S, BAba U (2005). Structural heart diseases in Nigerian children. Niger J Med.

[22] Ogah O, Adegbite G, Udoh S, Ogbodo E, Ogah F (2014). Chronic rheumatic heart disease in Abeokuta , Nigeria: Data from the Abeokuta heart disease registry. Nig J Cardiol.

[23] Jaiyesimi F, Antia A (1981). Childhood rheumatic heart disease in Nigeria. Trop Georg Med.

[24] Akpa M, Dodiyi-Manuel S, Agada Z, Odia O (2012). Rheumatic heart disease in Port Harcourt, Nigeria: clinical, demographic and echographic features. Port Harcourt Med J.

[25] Akintunde A, Opadijo O (2009). Late presentation of rheumatic heart disease: a justification for renewal of preventive methods. Pan Afr Med J.

[26] Kaplan E (2005). Pathogenesis of acute rheumatic fever and rheumatic heart disease: evasive after half a century of clinical, epidemiological and laboratory investigation. Heart.

[27] Essien I, Onwubere B, Anisuba B, Ejim E, Andy J, Ike S (2008). one year echocardiographic study of rheumatic heart disease at Enugu, Nigeria. Niger Postgr Med J.

[28] Sani MU, Karaye KM, Borodo MM (2007). Prevalence and pattern of rheumatic heart disease in the Nigerian savannah. an echocardiographic study. Cardiovascular Topics.

[29] Talwar KK, Gupta A (2016). Predictors of mortality in chronic rheumatic heart disease. Indian J Med Res.

[30] Mocumbi A (2012). The challenges of cardiac surgery for African children. Cardiovasc J Afr.

[31] Omokhodion S (2006). Management of patients with rheumatic fever and rheumatic heart disease in Nigeria-need for a National system of primary, secondary and tertiary prevention. S Afr Med J.

[32] Karthikeyan G, Mayosi B (2009). Is primary prevention of rheumatic fever the missing link in the control of rheumatic heart disease in Africa. Circulation.

